# Highly pathogenic avian influenza: pandemic preparedness for a scenario of high lethality with no vaccines

**DOI:** 10.3389/fpubh.2025.1613869

**Published:** 2025-07-16

**Authors:** Cristina Possas, Ernesto T. A. Marques, Alessandra Oliveira, Suzanne Schumacher, Marilda M. Siqueira, John McCauley, Adelaide Antunes, Akira Homma

**Affiliations:** ^1^Bio-Manguinhos, Oswaldo Cruz Foundation, Rio de Janeiro, Brazil; ^2^Aggeu Magalhães Institute, Oswaldo Cruz Foundation, Recife, Brazil; ^3^Department of Infectious Diseases and Microbiology, University of Pittsburgh, Pittsburgh, PA, United States; ^4^Chemical Industry Information System – SIQUIM, School of Chemistry, Federal University of Rio de Janeiro, Rio de Janeiro, Brazil; ^5^Laboratory of Respiratory Viruses, Exanthematous Viruses, Enteroviruses, and Viral Emergencies, Oswaldo Cruz Foundation, Rio de Janeiro, Brazil; ^6^Worldwide Influenza Centre, The Francis Crick Institute, London, United Kingdom; ^7^Academy of Intellectual Property and Innovation, National Institute of Industrial Property, Rio de Janeiro, Brazil

**Keywords:** HPAI, pandemic preparedness, mutation, spillover, vaccine governance, artificial intelligence

## Abstract

Highly Pathogenic Avian Influenza (HPAI) viruses, particularly H5N1 and H7N9, have long been considered potential pandemic threats, despite the absence of sustained human-to-human transmission. However, recent outbreaks in previously unaffected regions, such as Antarctica, suggest we may be shifting from theoretical risk to a more imminent threat. These viruses are no longer limited to avian populations. Their increasing appearance in mammals, including dairy cattle and domestic animals, raises the likelihood of viral reassortment and mutations that could trigger a human pandemic. If such a scenario unfolds, the world may face a crisis marked by high transmissibility and lethality, without effective vaccines readily available. Unlike the COVID-19 pandemic, when vaccines were rapidly developed despite inequities in access, the current influenza vaccine production model, largely reliant on slow, egg-based technologies, is insufficient for a fast-moving outbreak. While newer platforms show promise, they remain in early stages and cannot yet meet global demand, which alerts to the urgent need for accelerating vaccine and drug development, especially universal vaccines, next-generation vaccine platforms designed to provide broad, long-lasting protection against a wide spectrum of HPAI virus subtypes and strains. Here we propose a paradigmatic shift toward a more integrated, digitalized One Health surveillance system that links human, animal, and environmental data, especially in high-risk spillover regions. We underscore that Artificial Intelligence can revolutionize pandemic preparedness strategies, from improving early detection to speeding up vaccine and drug development and access to medical care, but should not be considered a stand-alone solution.

## Introduction

Infections with zoonotic influenza viruses, including Highly Pathogenic Avian Influenza (HPAI) viruses pose a significant threat to global public health in scenarios where viruses could acquire the ability to transmit efficiently among humans and cause a pandemic. Particularly concerning are the avian influenza strains, such as H5N1 and H7N9, which can be, transmitted from birds to humans, with high case fatality rates (1), even considering that most of H7N9 zoonotic infections result from LPAI (Low Pathogenic Avian Influenza) viruses and not from HPAI viruses (2, 3). In addition, avian influenza viruses can mutate or reassort, potentially leading to more transmissible or lethal variants. Although to date there is no evidence of sustained human-to-human transmission of avian influenza viruses, this possibility of mutation or reassortment is concerning and requires urgent global strategies for preparedness (4, 5).

Understanding the epidemiological behavior of avian influenza viruses, the mechanisms underlying zoonotic spillover, and the potential public health ramifications of a highly lethal pandemic scenario is imperative for global preparedness. From a knowledge governance perspective, this calls for the urgent implementation of robust, integrated surveillance systems that encompass poultry farm environments, sylvatic animals, and human populations with high exposure risk. These systems must be complemented by the development and equitable distribution of rapid, point-of-care diagnostic tools and by the strategic deployment of non-pharmaceutical interventions aimed at slowing viral transmission in the absence of immediate pharmacological solutions.

In addition, we highlight the importance of international collaboration, risk communication, and equity considerations in resource allocation during vaccine and drug shortages (6). By addressing the unique challenges of this worst-case scenario, the article aims to contribute to a more resilient global preparedness framework, supported by quality data and Artificial Intelligence, capable of managing unprecedented public health crises. In a previous publication we emphasized the critical need for urgent and sustainable investments in vaccine innovation and global preparedness. The results of our publication warn that a future pandemic caused by avian influenza viruses could unfold in the absence of effective vaccines, given current technological and logistical limitations (7).

Key barriers include restricted access to vaccine patents, reliance on slow and labor-intensive egg-based production methods, and the insufficient advancement and technological limitation of the current mRNA platforms and universal influenza vaccine technologies. These challenges are particularly alarming in the context of viral evolution and adaptation within farmed animals and their human handlers, which heightens the risk of zoonotic spillover and widespread outbreaks. This potential vaccine gap underscores the urgent need for a comprehensive global pandemic preparedness framework. Such a model must prioritize the integration of genomic and antigenic surveillance within a unified One Health approach, while also fostering robust public-private partnerships and scaling up investment in innovative vaccine platforms. These efforts are essential not only for controlling highly pathogenic and low pathogenicity avian influenza viruses but also for anticipating and mitigating other emerging zoonotic threats (8).

Our article underscores the need for developing a universal influenza vaccine to reduce the risk of future pandemics, advocating for stronger international coordination led by organizations like World Health Organization (WHO) and Pan American Health Organization (PAHO) to improve vaccine accessibility and efficacy. Universal highly pathogenic avian influenza (HPAI) vaccines refer to next-generation vaccine platforms specifically designed to provide broad, long-lasting protection against a wide spectrum of HPAI virus subtypes and strains. Unlike conventional influenza vaccines that require frequent updates to match circulating strains, universal HPAI vaccines aim to target highly conserved viral regions—such as the hemagglutinin (HA) stalk domain, internal proteins (like NP and M1), or T-cell epitopes—that are less prone to antigenic drift and shift. By focusing on these conserved viral components, universal vaccines have the potential to induce cross-protective immune responses, reducing the need for strain-specific reformulation and offering a more effective tool for pandemic preparedness and control of both known and emerging HPAI variants (9, 10). Such vaccine candidates may utilize diverse platforms, including recombinant proteins, viral vectors, or mRNA technologies, and are under active investigation in both animal and early-phase human studies. Universal HPAI vaccines represent a critical innovation pathway toward overcoming the logistical and scientific limitations of current egg-based or strain-specific vaccines, especially in rapidly evolving outbreak scenarios (11, 12).

In this article we examine the epidemiological dynamics of HPAI and LPAI viruses, zoonotic spillover pathways, and societal and healthcare implications of a highly lethal pandemic. We emphasize the necessity of robust One Health surveillance systems, innovative vaccine technologies, international collaboration, and the role of Artificial Intelligence (AI) in bolstering preparedness and response mechanisms.

### Epidemiological scenario: potential routes of zoonotic spillover

Avian influenza viruses, including HPAI viruses, are characterized by their ability to mutate rapidly, spreading and enabling them to adapt to new hosts. The primary zoonotic transmission routes include direct contact with infected birds and animals, exposure to contaminated environments, like faeces, etc., and the preparation of, and consumption of undercooked poultry (meat or other animal products). Further human activities, such as intensive farming and wildlife trade, amplify the risk of spillover events, events (by increasing the instances of human-animal interactions).

Avian influenza presents a critical global health threat, as indicated in Table 1. According to WHO, from January 1 2003 to December 12, 2024, 954 confirmed human cases of HPAI influenza A (H5N1) virus infection were reported across 24 countries, with 464 fatalities. Cases and fatalities related to other HPAI viruses are indicated in Table 1. While the global trend has been a decline in H5N1 human cases since 2015, recent reports (13, 14) indicate an increasing number of new human infections. For instance, as of January 6, 2025, the United States had reported 66 confirmed human cases of H5N1 since 2024, with one fatality. Historically, cattle were not considered natural hosts for H5N1, however recent cases (such as in the US in 2024) indicate that the virus can infect dairy cows, particularly spreading their mammary glands. In addition, in January 2025, a human case of H5N1 was detected in England, the second symptomatic case in the UK.

**Table 1:** Zoonotic avian viruses: main characteristics and key concerns.

Viral subtype	HPAI/LPAI primary hosts	Main outbreaks	Key concerns
H5N1	Poultry, wild birds, mammals (4) Worldwide, the detection of A (H5N1) viruses in non-avian species, such as marine and terrestrial mammals (both domestic and wild), has increased in recent years (47)	1997 (Hong Kong) (48); 2003-Present (global) (49)	Human infections (over 890 confirmed human cases and more than 460 deaths as of 2024) (1, 4, 28, 50), pandemic potential, increased transmission to mammals, risk of viral reassortment in mammals
H5N6	Poultry, wild birds (51) Aquatic poultry (ducks and geese) and migratory birds (52)	2014-Present (China, Southeast Asia) (53)	Sporadic human cases (at least 84 confirmed human cases and 33 deaths reported globally by 2024) (54), possible reassortment with other flu viruses (52)
H5N8	Poultry, wild birds (51)	2014-Present (Europe, Asia, Africa) (51, 55, 56)	Highly contagious in birds, occasional mammal infections (no confirmed human cases to date, but multiple mammal infections in seals and foxes reported) (57, 58)
H7N9	Poultry (59)	2013–2019 (China) (59)	Mutations increasing human infectivity, pandemic potential. Over 1,500 laboratory-confirmed human cases and at least 615 deaths reported during epidemics (59, 60)
H10N3	Poultry, wild birds (61, 62)	2021 (China) (63)	Limited human infections (only 1 confirmed human case as of 2024), needs monitoring (63, 64)
H9N2	Poultry, wild birds (65)	Ongoing (Asia, Middle East) (63, 65)	Reassortment potential with other flu viruses. More than 90 sporadic human cases reported with low case fatality rate (66)

Sources: elaborated by the authors based on Refs. (1, 4, 28, 47–66).

It is important to note that although human cases have been relatively low, H5N1 has been spreading extensively among bird populations and has recently been increasingly detected in several mammalian species, including dairy cows and wild animals. For example, in the United States, since 2022, the United States Department of Agriculture Animal and Plant Health Inspection Service has reported HPAI A (H5N1) virus detections in more than 200 mammals (15).

Emerging evidence suggests that certain HPAI strains, particularly from clade 2.3.4.4b, have acquired mutations that may enhance their capacity to infect mammals. Among these, changes in the PB2 gene—such as E627K and D701N—have been identified as key molecular markers that improve viral replication efficiency in mammalian hosts. Although these mutations have been detected in sporadic cases, particularly in mammals exposed to infected birds, there is no conclusive evidence of sustained mammal-to-mammal transmission to date. Nonetheless, the detection of such adaptations underscores the need for heightened genomic surveillance at the human–animal interface, where the potential for zoonotic spillover is greatest.

Another critical genetic determinant in the adaptation of avian influenza viruses to mammalian hosts is the PB2 gene, which encodes one of the three polymerase subunits essential for viral replication. Specific mutations in PB2, notably E627K and D701N, have been repeatedly associated with enhanced viral replication efficiency at the lower temperatures found in the mammalian respiratory tract (16). These mutations can significantly increase the virulence and transmissibility of HPAI viruses in mammals, including humans. Studies following experimental infections and epidemiological investigations of zoonotic cases have consistently highlighted PB2 mutations as key markers for assessing pandemic potential. The capacity of these mutations to facilitate cross-species transmission underlines the importance of incorporating PB2 surveillance into global risk assessment frameworks for avian influenza viruses.

The transition of H7N9 from low pathogenic avian influenza (LPAI) to highly pathogenic avian influenza (HPAI) is marked by the acquisition of a polybasic cleavage site in the hemagglutinin (HA) protein, leading to increased virulence in poultry (17). However, this change does not necessarily enhance transmissibility or severity in humans. Notably, both LPAI and HPAI H7N9 strains have been linked to severe human infections, with case fatality rates around 40% (18).

#### HA cleavage site and receptor binding specificity

While the polybasic cleavage site facilitates systemic spread in avian hosts by enabling HA cleavage by ubiquitous proteases, human pathogenicity is more influenced by receptor-binding specificity and host factors than by the cleavage site alone.

#### Residue 226 mutation and receptor affinity

The Q226L amino acid substitution in the hemagglutinin (HA) protein shifts receptor-binding preference from avian-type (*α*-2,3-linked sialic acids) to human-type (α-2,6-linked sialic acids), potentially increasing the risk of zoonotic transmission (19–21). Importantly, this substitution in the H7 HA does not eliminate affinity for avian receptors, allowing the virus to infect both avian and human hosts.

However, it is important to note that H1N1 viruses, including the 1918 pandemic strain and the 2009 H1N1 strain, do not follow this receptor-binding model. Structural and functional assessments of the 1918 virus indicate that its HA adapted for human transmission through distinct mechanisms rather than solely relying on the Q226L mutation (22). Previous studies, such as those by Gamblin et al. (23) have analyzed receptor binding affinity for the 1918 virus, providing insights into its human adaptation. Additionally, research on ferret-transmissible H5N1 viruses has examined similar receptor-binding changes, further informing our understanding of zoonotic transmission (24, 25).

#### Implications for zoonotic transmission

While the Q226L mutation may facilitate initial cross-species transmission, additional mutations are likely required for sustained human-to-human transmission. While the acquisition of a polybasic cleavage site is a defining feature of HPAI viruses and enhances their pathogenicity in birds, it does not necessarily correlate with increased or decreased zoonotic risk. Both LPAI and HPAI strains have demonstrated the capacity to cause severe disease in humans under certain exposure conditions. Continuous surveillance of these mutations is crucial to assess their impact on transmissibility and pathogenicity.

### Zoonotic risks and mortality rates

**Figure 1:**
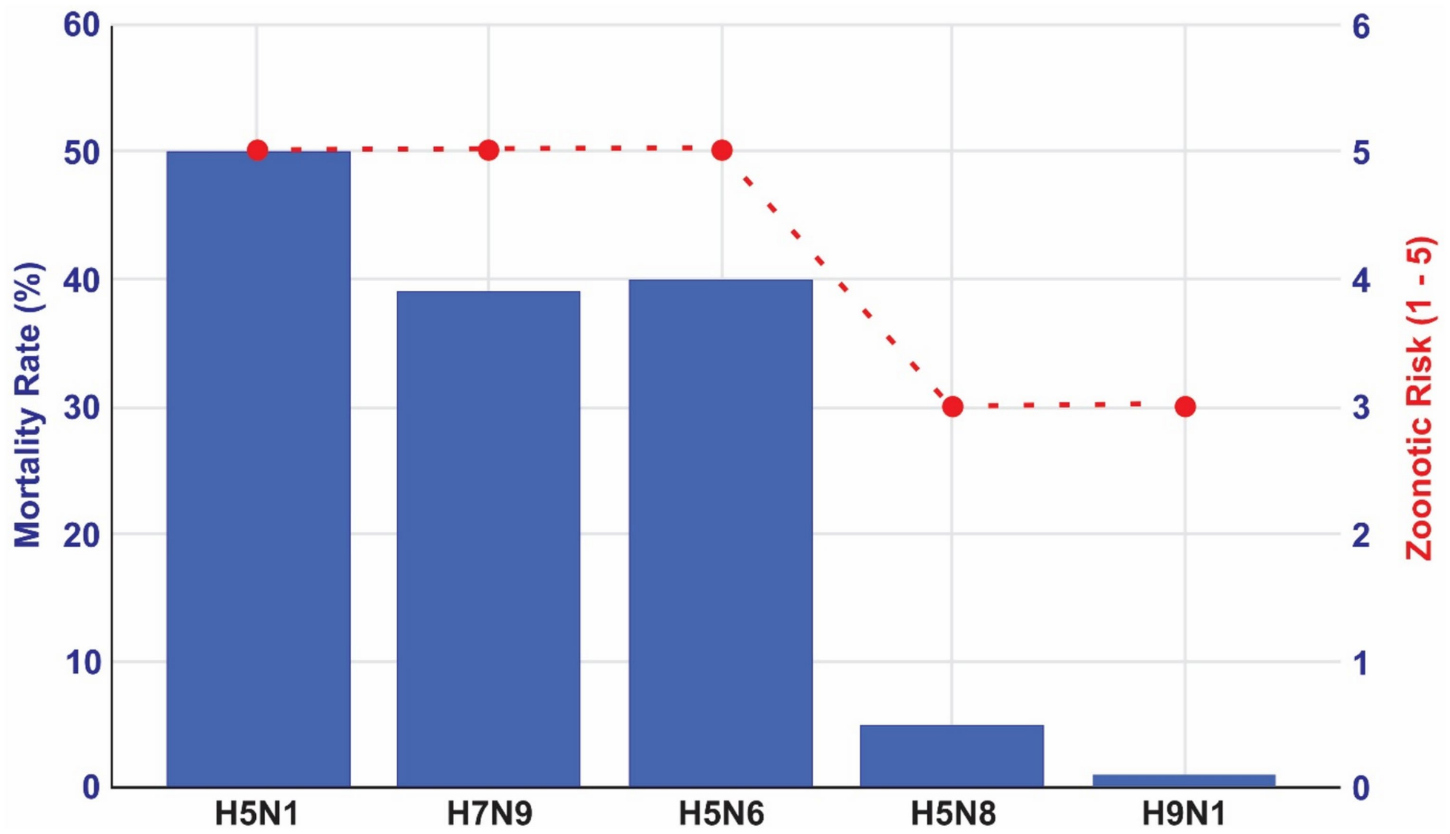
Zoonotic risks and mortality rates for the main HPAI viral subtypes. Source: elaborated by the authors based on World Health Organization (26) and Public Health Agency of Canada. Government of Canada (27).

In Figure 1, we compare the main high-risk HPAI subtypes considering zoonotic risks and mortality rates. The classification of zoonotic risk for HPAI viruses is based on factors such as the frequency and severity of human infections, potential for human-to-human transmission, and genetic characteristics of the virus. While there is not a universally standardized 1-to-5 scale, various health organizations assess these risks to inform public health responses.

The scores calculated in Figure 1 are scientifically based. We supported our classification of zoonotic risks based on two sources (26, 27). We supported our classification of zoonotic risks based on two sources, The Public Health Agency of Canada and WHO. The first one classifies certain influenza A virus subtypes, including H5N1, H5N6, and H7N9, as Risk Group 3 Human Pathogens due to their significant potential to cause serious human or animal disease (27). Similarly, WHO conducts risk assessments for specific HPAI and LPAI strains. In a 2022 assessment, the WHO evaluated the zoonotic risk of the A(H5N1) clade 2.3.4.4b viruses (28–30), considering factors like human cases, virus spread in wild and domestic animals, and genetic mutations. In Figure 1 we provide an indication of these zoonotic risks according to mortality rates, considering the possibility of overestimate of mortality rate due to underestimation of the number of infections especially.

These assessments, while not always presented on a numerical scale, provide a framework for scoring the relative zoonotic risks of different HPAI subtypes.

### Accelerating HPAI vaccine innovation and development: technological gaps

Innovative approaches to vaccine development are urgently needed and key points include addressing technological development and production challenges to overcome technological gaps. Current egg-based vaccine production is slow and difficult to scale in an emergency, especially during an HPAI virus outbreak that could lead to massive animal culling. While mRNA technology shows promise, questions remain about the duration, breadth of protection and scalability (31). Due to these constraints, our previous study (7) provided evidence indicating that vaccine development for HPAI is lagging, with very few active patents and limited advancements in universal vaccines. This scenario hampers the global capacity to respond effectively to a potential pandemic. Substantial investment in universal influenza vaccines is crucial to address the limitations of current technologies.

In addition to the technological barriers previously discussed, a critical challenge in HPAI vaccine preparedness is the antigenic mismatch between stockpiled vaccines and the strain that causes a future outbreak. This scenario is increasingly likely given the accelerated antigenic drift and shift observed in H5Nx viruses. For example, while clade 2.3.4.4b viruses currently dominate outbreaks in Europe and North America, regions of Asia are still reporting circulation of other clades such as 2.3.2 and 2.3.3. This geographic heterogeneity in dominant clades increases the risk that existing vaccine stockpiles, often produced against earlier strains, will have reduced efficacy if deployed during a novel outbreak elsewhere. Moreover, regulatory and manufacturing timelines for updating avian influenza vaccines lag behind the pace of viral evolution, further compounding this gap. This underscores the urgency for next-generation HPAI vaccines that offer broader cross-clade protection, such as those targeting conserved viral epitopes or using novel platforms like mRNA and recombinant technologies (32, 33).

### Broadly neutralizing antibodies: recent findings for potential universal flu vaccine

A recent breakthrough study by researchers from the University of Pittsburgh, in collaboration with the NIH Vaccine Research Center, demonstrated that monkeys pretreated with a moderate dose of the broadly neutralizing antibody MEDI8852^1^ were universally protected against HPAI viruses. In addition to confirming the antibody’s efficacy in preventing serious adverse health outcomes, the scientists established the minimum serum concentration required for protection in primate models (34) for further development of universal HPAI vaccines. For instance, deep learning approaches have been used to predict antigenic drift in H5N1 hemagglutinin variants, helping researchers anticipate viral escape mutations (35). Similarly, support vector machines and random forest algorithms have been applied to forecast epitope binding affinity and immunogenicity, enabling more targeted vaccine design (36). Recent studies have also demonstrated the use of neural network-based models to optimize mRNA vaccine sequences for enhanced expression and immunogenicity, which could be adapted for influenza vaccines in the future (37).

### Pandemic preparedness and AI: enhancing genomic surveillance, knowledge governance and sustainability

Artificial Intelligence (AI) has begun to reshape the landscape of pandemic preparedness, particularly by enhancing the speed and precision of epidemiological surveillance and knowledge governance. In vaccine development, machine learning models can predict antigenic properties, simulate immune responses, and optimize candidate selection, accelerating preclinical pipelines (38, 39). In parallel, AI-powered epidemic intelligence systems can process diverse unstructured data—from genomic databases to wildlife surveillance—to detect abnormal patterns and emerging threats before they affect human populations.

These advances are especially relevant for HPAI, where rapid viral evolution and zoonotic spillover require integrated early warning systems. AI algorithms can assimilate real-time inputs from genomic sequencing, environmental monitoring, animal health records, and social behavior to anticipate outbreak hotspots. This predictive capacity enables health authorities to issue early alerts and prioritize surveillance resources more effectively.

Artificial Intelligence (AI) is emerging as a revolutionary tool, reshaping public health with advancements in analyzing and preventing future pandemic scenarios. Additionally, AI supports robust genomic and antigenic surveillance. Genomic analysis allows tracking of mutations and reassortments that enhance virulence or transmissibility, while antigenic assays assess how well existing immune responses recognize evolving HPAI strains. Integrating both approaches is critical to guide vaccine updates and antiviral strategies.

Beyond surveillance, AI offers transformative applications across operational domains of pandemic preparedness. When adequately designed and integrated within resilient health systems, AI can significantly enhance outbreak forecasting, optimize allocation of medical resources, and support rapid diagnostics. For example, deep learning models have been deployed to predict regional outbreak hotspots based on climatic and migratory bird data, as demonstrated during H5N1 outbreaks in Southeast Asia (40). AI-driven decision support systems have also been used to optimize stockpiling and distribution of antiviral medications and personal protective equipment in real-time emergency settings (41). Additionally, AI-powered diagnostic tools using image recognition and molecular data processing have accelerated point-of-care detection of avian influenza strains in field conditions (42). These efforts must also be grounded in the One Health framework, requiring international coordination and data sharing across human, animal, and environmental health domains to ensure comprehensive risk assessment (7, 26).

### Designing AI to revolutionize pandemic preparedness

Artificial Intelligence (AI) is emerging as a revolutionary tool in public health, with its potential to analyze vast amounts of data, identify trends, and enable informed decision-making. For HPAI, AI could help identify conserved viral epitopes across multiple subtypes, guiding the rational design of universal or broadly protective vaccines. Recent studies have used machine learning models to predict conserved B-cell and T-cell epitopes in H5N1 and H7N9 hemagglutinin and neuraminidase proteins, accelerating preclinical evaluation of cross-protective candidates (35, 43). In pharmacological pipelines, AI-based virtual screening platforms have been applied to search large chemical libraries for molecules with predicted binding affinity to influenza polymerase and neuraminidase targets, significantly reducing lead compound identification time (6). Notably, deep learning frameworks have also been used to repurpose existing antiviral drugs against emerging HPAI strains by predicting off-target antiviral activities (44).

One of the most critical roles of AI is accelerating vaccine and drug discovery. For HPAI, AI can help identify conserved viral epitopes across subtypes, guiding development of universal or broadly protective vaccines. In pharmacological pipelines, it can screen large chemical libraries to identify potential antiviral compounds, reducing the time from discovery to clinical testing. Inclusive governance, characterized by equitable decision-making and transparent data sharing across countries and regions, is fundamental to effective global pandemic preparedness. This includes open access to viral genomic sequences, real-time epidemiological reporting, and collaborative use of AI-driven surveillance platforms to ensure timely detection and response to HPAI threats (45).

AI also facilitates strategic decision-making in resource-constrained scenarios. Algorithms can integrate epidemiological data, health system capacity, and demographic variables to prioritize vaccine allocation, deploy health workers, and anticipate regional surges in infection. Logistics systems enhanced by AI can ensure timely distribution of critical supplies such as PPE, antivirals, and ventilators, especially in underserved areas.

Moreover, AI can support integration of epizootic surveillance with immunization efforts. By linking real-time data from wildlife and livestock with mutation tracking, AI enables targeted containment and adaptive vaccination strategies. This is crucial to prevent the emergence of vaccine-resistant strains or hidden transmission pathways.

However, the success of AI depends on high-quality data inputs, inclusive governance, and ethical frameworks. It must be implemented as part of a broader transdisciplinary preparedness strategy, not as a stand-alone solution. AI’s greatest value lies in its ability to support rapid, data-driven action within a collaborative, globally coordinated response.

Beyond early warning and surveillance, AI also provides valuable tools for accelerating vaccine and drug development, optimizing resource-allocation, and integrating epizootic surveillance systems.

1. Accelerating vaccine and drug development

For HPAI, AI could help identify structurally conserved regions of viral proteins across multiple strains, supporting the development of vaccines that offer broad protection. In drug discovery, AI can analyze vast libraries of chemical compounds to identify potential antiviral candidates, drastically reducing the timeline from research to deployment.

2. Optimizing resource allocation

In a pandemic scenario marked by high lethality and scarce resources, AI supported by quality data could assist policymakers in making data-driven decisions about resource distribution. AI models could help a timely response to a broad range of indicators, such as population density, healthcare infrastructure, and disease transmission patterns to prioritize vaccine allocation, to deploy healthcare personnel, and to optimize hospital capacities.

AI-based logistics systems can predict areas likely to experience surges in cases, enabling timely delivery of critical supplies like personal protective equipment and ventilators. This proactive approach could ensure that even resource-limited regions are adequately supported.

3. Integrating epizootic surveillance and immunization

AI can play a critical role in supporting an integrated “big data” monitoring system that combines epizootic surveillance and immunization, if vaccines are not available. This integrated system can be a powerful tool to prevent and contain outbreaks, identifying viral circulation, monitoring mutations, and detecting early infections in domestic and wild birds. This information is essential for guiding targeted immunization programs and adjusting vaccine formulations to match emerging strains. Molecular diagnostics and genomic sequencing enhance the ability to track viral evolution, while international cooperation through organizations such as the World Organization for Animal Health (WOAH) and the Food and Agriculture Organization (FAO) facilitates data sharing and coordinated responses. Without comprehensive vaccine-oriented surveillance, immunization efforts may become ineffective due to the emergence of vaccine-resistant variants or undetected transmission routes. This AI strategy alongside stringent biosecurity measures and global cooperation, including geo-politically sensitive routes, is essential to mitigating the threat of HPAI and preventing future pandemics.

## Conclusion

Global epidemiological reports indicate that we might be entering a new era of Avian Flu, with the H5N1 strain spreading more rapidly among mammals. Although cases have been linked to infected wild birds and livestock farms, the virus is now spreading not only among birds and domestic animals, but increasingly infecting mammals.

Indeed, H5N1 viruses have been found in both wild and captive mammals, and they can sometimes cause fatalities as well as severe illness. Additionally, H5N1 detections in domestic cats are gaining attention. The US Department of Agriculture’s Animal and Plant Health Inspection Service reports that the HPAI H5N1 strain was found in a domestic cat in Colorado State on 2025/01/31 (15). Notably the B3.13 strain of the Eurasian 2.3.4.4b clade H5N1 virus has been spreading in animals not historically attributed as reservoirs for the HPAI virus (46). In relation to human infection, the World Health Organization (WHO) reported from 24 countries that between 2003 (beginning) and 2024 (2024/12/12), there were 954 human cases of H5N1, resulting in 464 fatalities, or 48.6% of the total zoonotic cases from avian influenza viruses (26).

A possible extreme scenario, in which a mutated strain becomes highly transmissible among humans, would create a global health crisis marked by significant morbidity and mortality. Preparing for a potential HPAI pandemic requires a multifaceted transdisciplinary approach that addresses epidemiological, technological, and societal challenges.

The possible absence of an effective HPAI vaccine for human in a highly lethal pandemic scenario, contrasting with rapid vaccine development in the COVID-19 pandemic, highlights the urgency of accelerating investment in innovative solutions and equitable global strategies. By leveraging AI strategies, fostering international collaboration and strengthening innovation funding mechanisms, the global health community can build a more resilient and sustainable innovation governance system capable of responding to unprecedented crises.

What is needed is a shift toward faster action and a coordinated, inclusive strategy that prioritizes preparedness before a next pandemic begins. The time to act is now.

## Data Availability

The original contributions presented in the study are included in the article/supplementary material, further inquiries can be directed to the corresponding author.

## Footnotes

^1^MEDI8852 is a fully human monoclonal antibody that targets the highly conserved stem region of influenza A hemagglutinin (HA), offering robust protection in preclinical models of H5N1and H7N9, even when administered up to 72 h post-exposure, and outperforming oseltamivir in key measures of survival and disease severity. It neutralizes all 18 subtypes of influenza A, including both Group I (H5N1) and Group II (H7N9) strains (67).
